# Monitoring the Production Information of Conventional Machining Equipment Based on Edge Computing

**DOI:** 10.3390/s23010402

**Published:** 2022-12-30

**Authors:** Yuguo Wang, Miaocong Shen, Xiaochun Zhu, Bin Xie, Kun Zheng, Jiaxiang Fei

**Affiliations:** 1 School of Automobile and Rail Transit, Nanjing Institute of Technology, Nanjing 211167, China; 2 Advanced Industrial Technology Research Institute, Nanjing Institute of Technology, Nanjing 211167, China; 3 Sino-German Intelligent Manufacturing Research Institute, Nanjing 211899, China; 4 Nanjing Kangni Precision Mechanics Co., Ltd., Nanjing 210038, China

**Keywords:** current signal, calibration, edge computing, real-time monitoring, operational status

## Abstract

A production status monitoring method based on edge computing is proposed for traditional machining offline equipment to address the deficiencies that traditional machining offline equipment have, which cannot automatically count the number of parts produced, obtain part processing time information, and discern anomalous operation status. Firstly, the total current signal of the collected equipment was filtered to extract the processing segment data. The processing segment data were then used to manually calibrate the feature vector of the equipment for specific parts and processes, and the feature vector was used as a reference to match with the real-time electric current data on the edge device to identify and obtain the processing start time, processing end time, and anomalous marks for each part. Finally, the information was uploaded to further obtain the part processing time, loading and unloading standby time, and the cause of the anomaly. To verify the reliability of the method, a prototype system was built, and extensive experiments were conducted on many different types of equipment in an auto parts manufacturer. The experimental results show that the proposed monitoring algorithm based on the calibration vector can stably and effectively identify the production information of each part on an independently developed edge device.

## 1. Introduction

With the current advancements in the Internet of Things, edge computing, big data, and other new-generation information technologies [1,2,3,4], as well as the demand for a personalized product and shorter delivery cycles, businesses are under intense competitive pressure, which necessitates the use of smart manufacturing to lower costs and boost efficiency [5]. In the machinery manufacturing sector, there is a huge number of offline equipment whose production data and processes need to be collected and monitored as traditional factories are upgraded to smart factories [6]. This allows enterprise managers to handle the production progress and make management decisions quickly and effectively [7]. Therefore, to address the offline equipment production monitoring problem faced by traditional enterprises, it is necessary to come up with a generalized and low-implementation cost equipment monitoring method with the help of current acquisition, edge storage, and edge computing technologies.

Compared to mechanical signals, such as acoustic signals [8], acceleration [9], vibration signals [10], and cutting forces [11], current signals [12] can better reflect information about the machining process of a part. Hence, the use of current sensors is an effective means to realize the monitoring of equipment. Unlike the current sensor-based device monitoring method, some researchers use the data read through the equipment’s own interfaces for analysis, including the Ethernet interface, RS485 serial port, RS232 serial port, and others [13,14,15,16,17]. However, since there is a large number of equipment from numerous different manufacturers in the shop, with many types and often non-compatible communication interfaces and protocols that make data collection through the interfaces challenging, expensive, and often even impossible, we decided to adopt an acquisition scheme based on current signals in this paper.

Currently, electrical signals-based equipment condition monitoring is mainly applied to fault diagnosis [18,19,20], tool wear [21,22,23], and energy consumption analysis [24,25,26,27] and is not massively applied in production management [28]. By modeling the relationship between the equipment conditions and data characteristics, researchers have achieved status identification in the monitoring process and further acquired information on fault, tool, and energy consumption, which helped the research on production management. However, the described scheme does not provide a solution strategy for high-frequency sampling, massive data storage, and long-time stable monitoring.

The equipment monitoring process can generate a huge amount of raw data. Edge computing can effectively solve the data storage problem by building an edge end at the data source to store a huge amount of data locally and extract only useful information from it for uploading. Edge computing has the benefits of real-time and reliability while also sharing the storage and computational strain of the central server [29,30]. However, due to the limited storage capacity and computational capacity of edge devices [31], the designed monitoring method must maintain long-term, stable performance under this premise.

Due to manufacturing differences, each device has its own unique characteristic, which is considered to be its RF fingerprint [32]. A similar phenomenon exists in the study of this work, where the same type of equipment processing and the same type of part generates different current waveforms. Therefore, the study of the RF fingerprint identification method has reference significance for this paper. On the other hand, it is difficult to obtain sufficient abnormal data because the anomalous state of the equipment is not easily reproducible. Recently, research on RF fingerprint recognition has made some progress using deep learning enhancement algorithms in the absence of sufficient training data.

The real-time monitoring of conventional machining equipment must meet the requirements of high adaptability, mass storage, high-frequency sampling, and steady monitoring, and obtain the production time information and abnormal information of parts. This paper proposes a production status monitoring method for traditional machining equipment based on edge computing and builds a prototype system. The edge end is situated at the location of the production equipment in the shop and uses current sensors to gather the total current of the equipment. It then creates a feature model by examining the correlation between the features of the data and the equipment’s operational status and uses the model to continuously track the equipment’s operational status. The collected electric current data are saved in the local database of the edge system, and the calculated production information is uploaded to the server.

It can identify the status of turning, milling, drilling, and boring processes, including power off, processing, tool change, anomaly, and other states. The high-frequency data acquisition and monitoring demands can be satisfied using the proposed monitoring method, which lowers the computing demands on embedded devices. It can count employee hours and compute employee performance automatically based on the processing time of the equipment.

This paper is organized as follows: Section 2 introduces the work related to monitoring by using a device interface, monitoring by using device current, and the monitoring framework by using edge computing. Section 3 introduces the edge monitoring framework of this paper. Section 4 describes the monitoring methods in this paper. In Section 5, the method is experimentally verified. Finally, this paper summarizes the research content and prospects for future work.

## 2. Related Work

Nunzio M. Torrisi et al. [15] developed the Cyber OLE for the Process Control (CyberOPC) monitoring system based on the OPC protocol. Gong Tao et al. [16] used acquisition devices such as the Programmable Logic Controller (PLC) and Distributed Control System (DCS) in the Supervisory Control and Data Acquisition (SCADA) platform to complete the data acquisition of the equipment. Kavianipour et al. [17] designed and implemented a data communication function based on the PCIe interface on Xilinx VC707 Virtex-7.

Miao et al. [19] extracted sensitive feature information from a large amount of historical condition data, combined digital twins to model the operational process state and effectively predicted the operational status of the production equipment to achieve fault warning. Li et al. [22] distinguished the machine tool stop, standby, start-up, no-load, and processing states based on parameters such as machine tool internal spindle power and monitored the tool wear conditions by comparing dynamically updated cutting power thresholds with real-time cutting power. Hu et al. [25] divided the machining equipment’s spindle running states into the startup, no-load, and cutting, and the state of the equipment was identified based on the spindle power value. An online system for tracking energy use was created as a result. Li et al. [26] divided the machining equipment conditions into four operational statuses: standby, no-load, processing, and start-up, and proposed a condition monitoring method based on recursive analysis and image processing techniques, and achieved part identification, condition identification, and energy efficiency analysis. Chen et al. [27] identified the shutdown, standby, no-load, and processing states of the machine tool based on the change characteristics of the total power of the tool. Then, they calculated the power consumption of each state. Finally, they created an energy efficiency monitoring and management system to cut energy consumption.

Liu et al. [33] developed a tool-monitoring edge system based on the calibrated spindle torque to monitor the process of tool wear during repetitive machining. By performing a simple similarity analysis between the characteristic signals generated by the reference tool during calibration and those generated by the monitored tool, the goal of full coverage monitoring of the shop floor machine was achieved. Wang et al. [34] developed an embedded edge-sensing computing node. By collecting and analyzing the motor current envelope signals at the computing node, they were able to perform equipment fault diagnosis, prediction, and maintenance. Syafrudin et al. [35] set up an edge system on an automotive assembly line to obtain environmental data from sensors such as gyroscopes and accelerometers for processing and rapid fault identification. Petrali et al. [36] used edge computing to analyze the sorter status in real-time and intelligently controlled the conveyor belt by sharing statuses to improve sorting efficiency and accuracy. Zhe et al. [37] developed an intelligent monitoring system for underground coal mines, which shortened the response time and significantly improved safety by reducing the transmission link to the cloud center. Hu et al. [38] described an intelligent production line by introducing edge computing. Their method expanded the computing resources and storage capacity of the cloud platform to the Internet of Things (IoT) edge and improved the efficiency of resource scheduling. Harmatos et al. [39] presented the benefits of the joint use of 5G Non-public Networks (5G-NPN), Time-sensitive Networking (TSN), and edge computing for manufacturing and the challenges of integrating these technologies. Taking a platform as an example, they verified the importance of deep connections from the network end to the edge end.

The reference [40] investigated the effect of environmental changes on the effectiveness of RF fingerprint identification and the problem that it is not easy to collect samples from different environments; the tapped delay line and clustered delay line (TDL/CDL) models were trained to improve the accuracy of recognizing transmitters significantly from 74% to 87.94% on the unobserved data. The reference [41] optimized the solution for the reference [40] and proposed a fine-grained augmentation approach to improve the learning performance of the deep learning model, resulting in an accuracy of 96.61% for recognition.

The references [15,16,17] interface-based monitoring method is not suitable for conventional off-line machines. The methods described in the references [19,22,25,26,27] for current-based monitoring are rarely employed to acquire production data. According to the research in references [33,34,35,36,37,38,39], edge computing has been more frequently employed for equipment monitoring. The references [40,41] show that deep learning augmentation algorithms have better recognition results in the case of insufficient training data. It is critical to developing a current-based edge monitoring approach with solid adaptability, cheap costs, and high reliability to suit the real-time monitoring needs of traditional processing equipment.

## 3. System Framework and Problem Definition

**Figure 1:**
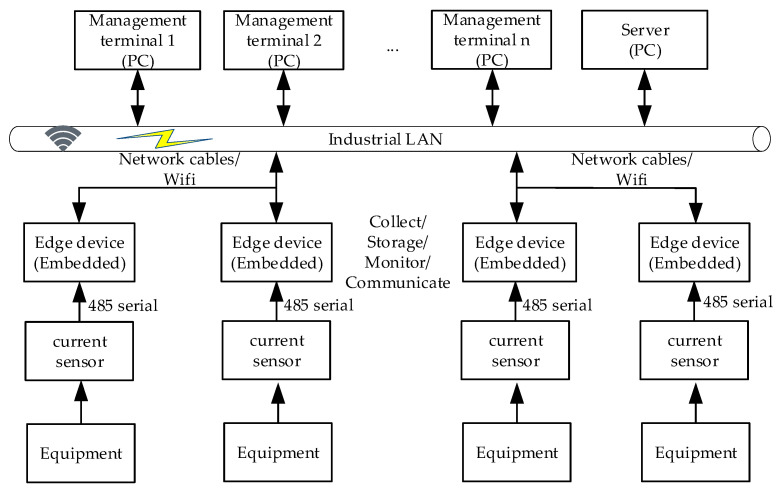
Block diagram of the overall composition of the equipment production status monitoring system consisting of current sensor, edge device, management terminal, and server.

The overall architecture of the equipment production status monitoring system is shown in Figure 1. The edge device receives the equipment current signal collected by the sensor through a 485 serial port. The data were then analyzed timely to obtain the production information of the workpiece. Next, the information was uploaded to the server through the network. Finally, the software of the management end obtains the production information for display and makes relevant production plans. The edge-side software was developed on a Linux system using C language. The software includes data acquisition, real-time monitoring, database storage, network transmission, and other functions.

The following three issues are what we needed to resolve in this paper based on the system architecture: how to implement the traditional processing equipment’s edge data acquisition and storage, how to define and calibrate the production features based on the collected current signal, and how to implement the real-time monitoring of equipment production information based on production features at the edge.

## 4. Real-Time Monitoring Method

For real-time status monitoring, a calibration vector-based monitoring method was adopted in this paper. Firstly, the start-up threshold, completion threshold, and standby threshold were manually chosen to obtain the processing status data. A section of the data was then used to calibrate the feature point, its range threshold, the feature interval, and its range threshold. The coordinates or range thresholds were then adjusted to ensure that the remaining sections of the process data fell inside the predetermined range entirely. The calibrated feature vectors were then uploaded to the process feature database to be stored.

In real-time monitoring, the feature signals of the process were first acquired. Then, based on the recognition algorithm, the start-up state of the equipment was identified. The feature intervals and points were then evaluated one by one sequentially. For machining, records that fall outside the calibration range are indicated with an alert prompt. Finally, the recognition process was complete, and the part’s start and end times, as well as the anomalous marks, were obtained.

### 4.1. Analysis of Equipment Operational Statuses

**Figure 2:**
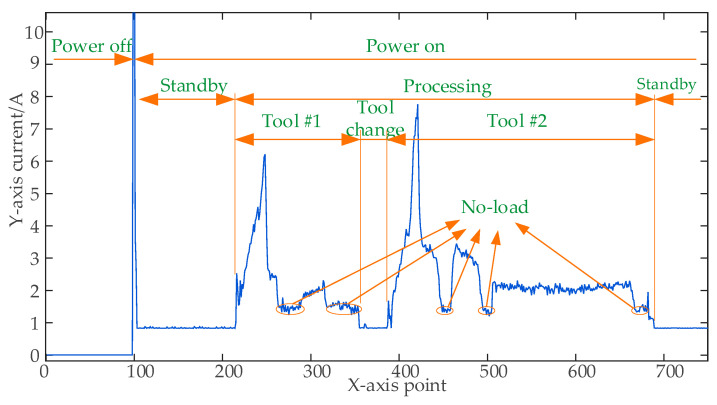
Current variation in a lathe under different operational statuses.

Using the computer numerical control (CNC) turning process of an automotive shaft as an example, we analyzed the relationship between the total current characteristics of the milling machine and the operational status. The total current of the running machine can be divided into a high level and a low level, corresponding, respectively, to the processing state and standby state of the machine tool. In the standby state, the current is steady, with occasional small fluctuations, and the standby current magnitude is recorded as *I_std_*. Compared with the standby state, the current fluctuates drastically in the processing state, with some peaks of *I_max_*, and the overall processing current remains between *I_std_*~*I_max_*. Between the standby and processing states, there are two transient states: start and finish. The data curves show a change in the current value from a low to a high level with spikes and from a high to a low level, respectively. The processing state can be divided into cutting, tool change, and no-load states. The cutting current *I_cut_* is high, the tool change current *I_ct_* is close to the standby current, and the no-load current *I_nol_* is between *I_cut_* and *I_ct_*. At the instance that the machine is turned on, the data curve shows an instantaneous rise and fall, and the peak current *I*_on_ is larger. During the normal operation of the machine, the relationship of the currents of different states is as follows: 0 < *I_std_* = *I_ct_* < *I_nol_* < *I_cut_* < *I_on_*. In addition to the difference in the magnitude of the currents when the equipment is in different states, there is also a time difference, mostly because the no-load period is shorter than the tool change time, and the tool change time is shorter than the standby time. Other types of conventional machining equipment have a similar relationship in the manufacturing process when defining the aforementioned criteria, as shown in Figure 2.

However, machine tools may encounter unusual circumstances in the machining process, such as operator error, equipment breakdown, tool wear, and subpar machining blanks. These anomalies will appear as changes in currents, including an extremely high current caused by tool collision, increases in the overall current or shortened processing time due to high magnification, a high current due to tool wear, and no load due to tool setting errors or secondary machining.

### 4.2. Time-Series-Based Feature Calibration Method

For traditional machine equipment, the machining process can be affected by such factors as voltage fluctuation, the surface quality of the part, the wear of the cutting tool, and improper operator actions; as a result, there may be discrepancies in the electric current data even when machining the same type of parts with the same equipment. To differentiate whether the parts are processed properly, an appropriate threshold range for the pertinent calibration parameters should be established to obtain the parts’ production data. The anomalous data waveform and the workpiece’s actual processing state are combined to determine the anomalous reasons. The specific calibration procedure is shown as follows.

Step 1: Choose a legacy data file, and filter the raw data with a Gaussian filter to eliminate the interference of random noise and obtain the filtered data without changing the original features.

Step 2: Select a section of the filtered data with a default size of 10,000 data points that contains processing data of multiple parts. Based on the characteristic behavior that the current value of the equipment increases monotonically to a certain value when the machine starts on the part, the start-up threshold *I_start_* can be determined manually. According to the current value stabilized at a certain value after equipment processing, the completion threshold *I_end_* can be determined manually. The standby threshold *T_wait_* can be determined by the fact that the tool change time is shorter than the standby time.

Step 3: Traverse the filtered data and determine whether the current value is less than *I_end_*; if it is, record the point sequence as *x_ws_* and start counting *num*++ until the current value is greater than *I_start_*. Judge whether *num* is greater than *T_wait_*; if so, record the standby start point sequence to the set {*pws*} and the endpoint sequence to the set {*pwe*}. Adjust the correspondence relationship of the sets to obtain the processing start point and processing end point of all the parts in between to achieve the separation of the machining state and standby state. 

Step 4: According to the above methods, the data of the processing state of each part are extracted, and the processing time and average current within the processing time of each part are calculated. The data of the anomalous state are removed, and the characteristic parameters are determined, including the processing period *T_work_* and standard average current *I_avg_*.

Step 5: In order to effectively identify the completion state of the equipment and to avoid the misidentification caused by current fluctuation around the completion threshold *I_end_* at the time of completion, the characteristic parameter standby variance *VAR* is calibrated according to the feature that the standby current is steady and the variance is close to zero. The parameters are then combined to obtain the feature vector *I_feature_* = {*I_start_*, *I_end_*, *T_work_*, *T_wait_*, *VAR*, *I_avg_*}. 

Step 6: Select one segment of the data for the part as the standard and plot the data in a curve form. Select local maximum points {*x_i_*, *y_i_*} on the curve as feature points, and set the threshold range of the direction and *y* direction of point *x* as *thr_xi*, *thr_yi*. Calculate the number of points in the range [*x_i_* − *thr_x_i_*, *x_i_* + *thr_x_i_*] [*y_i_* − *thr_y_i_*, *y_i_* + *thr_y_i_*] for the rest segments of the processed data. If the number of points is greater than zero, it means the set range is appropriate; if not, reset the range value. Save the feature point vector *I_point_i_* = {*x*, *y*, *thr_x*, *thr_y*} (*i* = 1,2…).

**Figure 3:**
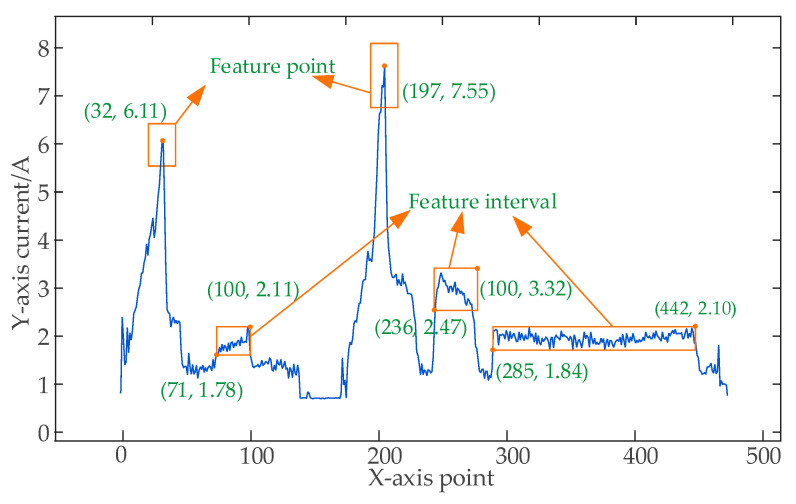
Schematic diagram of feature vector calibration.

Step 7: Select processing intervals [*x_left_*,*x_right_*] on the curve as monitoring intervals, and set the upper and lower limits *thr_up*, *thr_down* of the interval in the *y* direction. Calculate the number of points thr_num in the range of intervals [*x_left_*, *x_right_*] [*y_left_* − *thr_down*, *y_left_ + thr_up*]. Determine whether or not the number of points in this interval for the remaining segments of the processed data is greater than 90% *thr_num*; if so, it means the range is set appropriately; if not, reset the range value. Save the feature interval *I_interval_j_* = {*x_left_*, *x_right_*, *y_left_*, *thr_up*, *thr_down*, *thr_num*} (*j* = 1,2…). Figure 3 displays the calibrated feature vector, where the red and blue boxes represent the feature point and interval range, respectively.

### 4.3. Constant Threshold Monitoring Method Based on Calibrated Feature Vector

During the start-up transient of the equipment, a high current is generated due to the tool’s collision with the workpiece. The trend is that the current increases monotonically, and its value will pass through the start-up threshold point. Additionally, the equipment is in a standby state before the current rises, the current is steady, and its value is typically lower than the *I_end_*. Thus, the start status can be identified. In addition, the current levels, before the machine finishes processing, and the electric current becoming steady, the variance per *T_wait_* data points moves closer and closer to zero. Hence, the completion status can also be identified. By checking if the characteristic points and characteristic intervals are within the predetermined range, any anomalous processing circumstances that may exist can be recognized. The pseudo-code of real-time monitoring is as Algorithm 1.
**Algorithm 1**: Pseudo code of real-time monitoring.**Input**: Current data set ***list***, counting variable *timeL* and *TL*, feature vector *I_feature_* = {*I_start_*, *I_end_*, *T_work_*, *T_wait_*, *VAR*, *I_avg_*}, feature point set ***I_point_i_*** = {*x*, *y*, *thr_x*, *thr_y*}, feature interval set ***I_interval_j_*** = {*x_left_*, *x_right_*, *y_left_*, *thr_up*, *thr_down*, *thr_num*}.**Output**: Vector ***S*** and ***E***, vector ***TimeS*** and ***TimeE*** //the start and end of a processing tool *Ab_pcs_* and *Ab_endw_*. //anomalous marks**while *true* do** //Automatic cycle monitoring of edge equipment after power on     Update *timeL* and ***list*** with sample data.    **1**: The raw data ***list*** is processed using real-time Gaussian filtering.    **2**: Identify the machine’s instantaneous start-up state by *I_start_*, *I_end_*, and *T_wait_*.         *TL*++ //*TL* is the number of processing data.         ***S.***
*push_back* (*timeL*-*TL*) // *timeL* is the number of collected total data.         ***TimeS.***
*push_back*(current time)    **3**: **if** *TL* > 0 **then**         **for** (i = 0; i< *length* (***I_point_***; i++) **do**             **if** i = = 0 **then**                Verify whether the startup is properly identified. If so, record the start time. If not, **goto 2** and reset the parameters. (*TL* = 0, ***S.***
*pop_back()*,***TimeS.*** *pop_back()*)             **else** Verify whether the feature point***I_point_*** falls within the preset range. If it is, feedback processing progress (*TL*/T*_work_*) %. If not, Indicator light alarm.             *Ab_pcs_* = false //The record is marked as an anomaly.             **end if**         **end for**
        **for** (j = 0; j < *length* (***I_interval_***; j++) **do**            Verify whether the feature interval***I_interval_*** falls within the preset range. If it is, feedback processing progress (*TL*/*T_work_*) %. If not, indicator light alarm.             *Ab_pcs_* = false //The record is marked as an anomaly.         **end for**    **4**: **if** *timeL* > = *T_work_ +T_wait_*_,_ **then**        Identify the machine’s instantaneous completion state by *T_wait_*, *VAR*, *I_avg_*.        Calculate the variance *var* of the ***list*** [*length*(***list***)-*T_wait_*, *length*(***list***)].         **if** *var* < *VAR*
**then**            Calculate a value *Tback* to adjust the completion point.             ***E.***
*push_back* (*timeL*-*T_wait_*-*Tback*).             ***TimeE.****push_back*(current time)             Calculate the average value *avg* of the ***list***[*length*(***list***)-*TL*, *length*(***list***)-*T_wait_*-*Tback*].             **if** abs(*avg*- *I_avg_*) > 0.2 **then**                *Ab_endw_*= true //The record is marked as an anomaly.             **end if**            Reset parameters. (*TL* = 0, *l**ist***.*clear*())        **end if**    **end if****end while**

## 5. Experimental Verification

In this section, we verify the accuracy and stability of the system developed based on the above monitoring methods when it is used in the workshop.

### 5.1. Production State Identification Experiment

**Figure 4:**
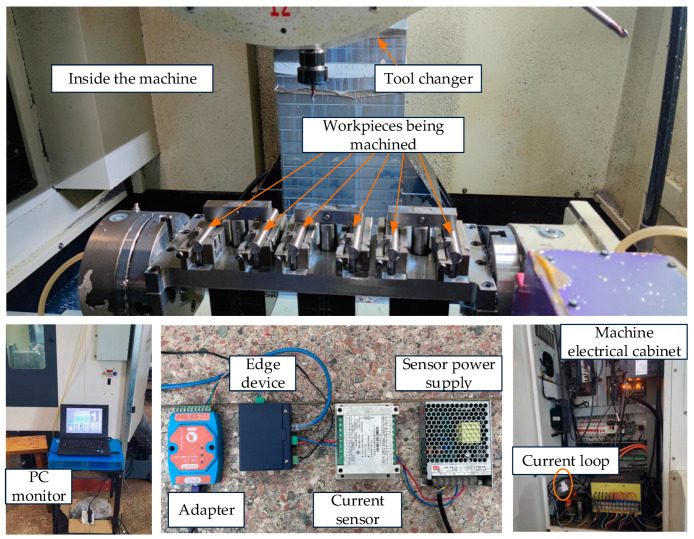
Experimental field equipment.

An edge device field monitoring device, including a current ring, current sensor, power supply, embedded chip, and experimental monitoring computer, is shown in Figure 4. Among them, the input range of the sensor is 400 V × 100 A, and the range/aperture of the external current ring is 0~100 A/16 mm. The capacity of the embedded storage chip at the edge is 64 GB, which can meet the monitoring storage demand for 128 days, and the execution time of the edge computing algorithm is less than 0.01 s, which can maintain a stable acquisition for a long time at 15.6 Hz.

### 5.2. Experimental Results and Analysis

**Figure 5:**
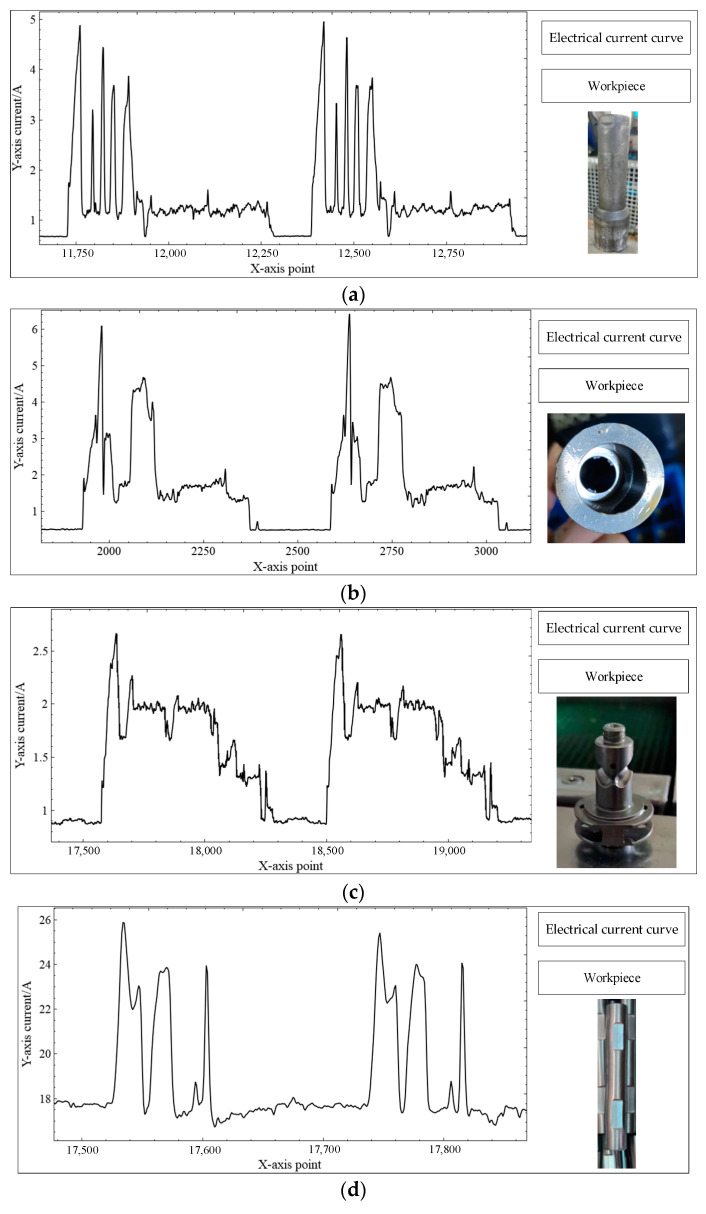

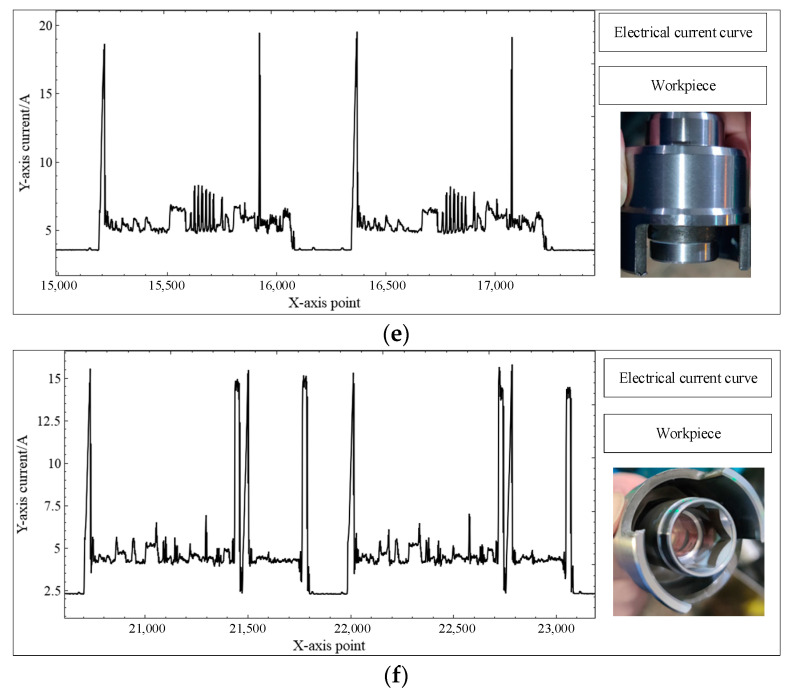
Photos and machining electric current curves of the experimental parts: (**a**) Toolholder—Turning small end bore; (**b**) Toolholder—Boring big end bore; (**c**) DriveShaft—Milling Ballways; (**d**) DiffCrossShaft—Forging; (**e**) HUB—Rough turning of end face outer and inner holes; (**f**) HUB—Fine turning of end face outer and inner holes.

At the workshop site, we conducted numerous tests; these tests mostly focused on the batch processing of multiple automotive parts using CNC equipment with reliable processing cycles involving lathes, milling machines, forging machines, and combination lathes. The processing cycle of the part process is constant. The loading and unloading methods for the blanks include automatic loading and unloading by robots and manual loading and unloading. A total of 38 sets of data were used in the experiment, and six sets of them were calibrated; the calibration results are shown in Table 1, and the corresponding photos and machining electric current curves of the experimental parts are shown in Figure 5. The part diagrams of Figure 5 were taken before and after machining, and the curves are current timing curves. Each graph contains two complete machining processes.

Take the first record in Table 1 as an example. The part name is a tool holder, and it is loaded and unloaded manually. It is used for the operation of turning small end inner holes by the lathe. Its *I_start_* and *I_end_* are 1.2 A and 0.8 A, respectively, and its *T_wait_* is 100, about (100/15.6) 6.4 s. Its *T_work_* is 546, and the processing time for a single part is (546/15.6) 35 s. Finally, the average value of this set of processing segment data (*I_avg_*) is 1.64 A.

As an illustration, the first record in Table 2 shows that there are 74 identified parts, the longest machining time for all the parts is 35.12 s, the shortest is 34.51 s, the longest waiting time is 24.94 s, the shortest is 7.01 s, and the average waiting time is 13.80 s. The actual quantity of the machined pieces, the length of the operation, and other data are recorded and compared with the outcomes of the system recognition provided in Table 2. The system offers a high recognition rate for the processing status of the equipment, regardless of manual or automatic loading and unloading. As shown in Table 2, the processing time for the different pieces of one type of part is practically the same, with a deviation of no more than 2 s. Parts 3 and 4 are automatically loaded and unloaded, so the standby time is essentially the same. Manual loading and unloading are used for the remaining components, and the standby time is different. The calculated average standby time for manual loading and unloading is of reference value for the advanced scheduling of production planning. The information on processing times acquired automatically can be used to calculate employee production performance in a more precise and equitable way. The standby time data that was acquired can help refine the production plan.

**Figure 6:**
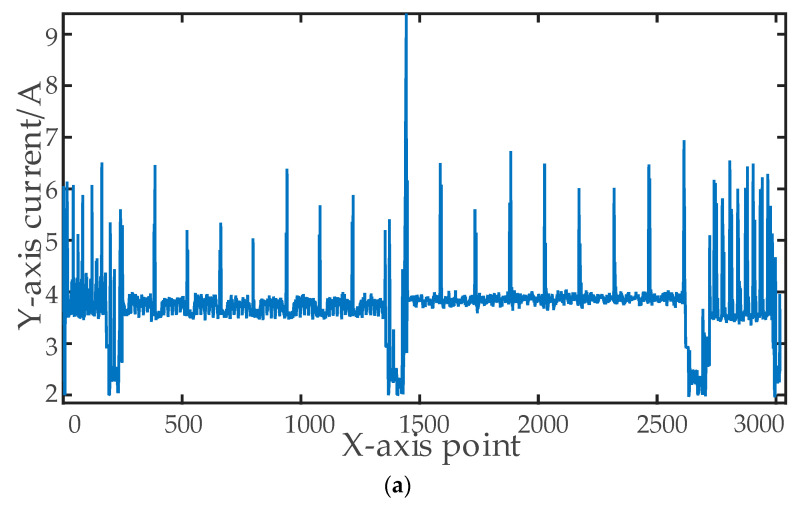

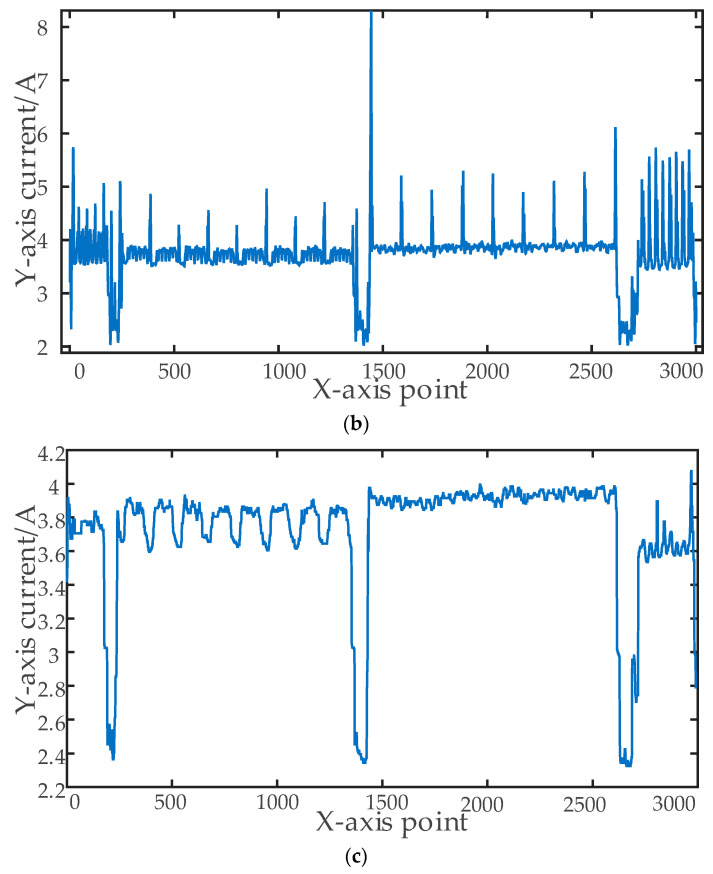
Curve comparison schematic of (**a**) Raw data curve; (**b**) Gaussian filtering curve; (**c**) Median filtering curve.

Take the batch milling of one of the shaft parts as an example: the processes are punching, chamfering, and milling grooves. At first, the legacy data collected from the part are filtered, and a comparison of the raw data and the filtered curve is shown in Figure 6. It shows that Gaussian filtering can reduce burrs while preserving the characteristics of the raw data, whereas median filtering distorts the original curve. Then, the filtered curve is segmented by the calibrated start threshold, finish threshold, and standby threshold, and the main feature vector is calculated to be (3.10876, 2.37219, 100, 3003, 0.2, 3.72957). Finally, one segment of the data was selected to manually calibrate the features, and the calibrated feature point vectors are shown in Table 3. Then, monitoring experiments were conducted at the workshop site in time slots, and some production records were taken as examples, as shown in Table 4. Further analysis yielded the average processing time of each part as 196.2 s, the longest processing time as 197.2 s, and the shortest processing time as 196.2 s, with a deviation of 2 s. The experiments show that the system has stable sampling and high accuracy.

### 5.3. Analysis of Anomalous States

Due to the complexity of processing, there can be various anomalies in the processing of workpieces, which are associated with the quality of workpieces, such as inaccurate dimensions and substandard surface quality. The system identification results were compared with the feature vector to identify data anomalies. By analyzing the electric current data, the processing data that were clearly different from the standard curve were captured and further verified in the shop to correlate with the actual production anomalies. In this experiment, if the processing time exceeded the standard processing time by more than 2 s, then it was judged that an anomaly existed in the machine multiplier. If the average current of the machined workpiece differed from the standard average current by more than 0.1 A, then it indicated a minor anomaly. If the current difference exceeded 0.2 A, it indicated a serious anomaly. The acquired anomalous marks can be combined with the processing time information to quickly locate the equipment abnormality, solving the problem in time and reducing the loss.

**Figure 7:**
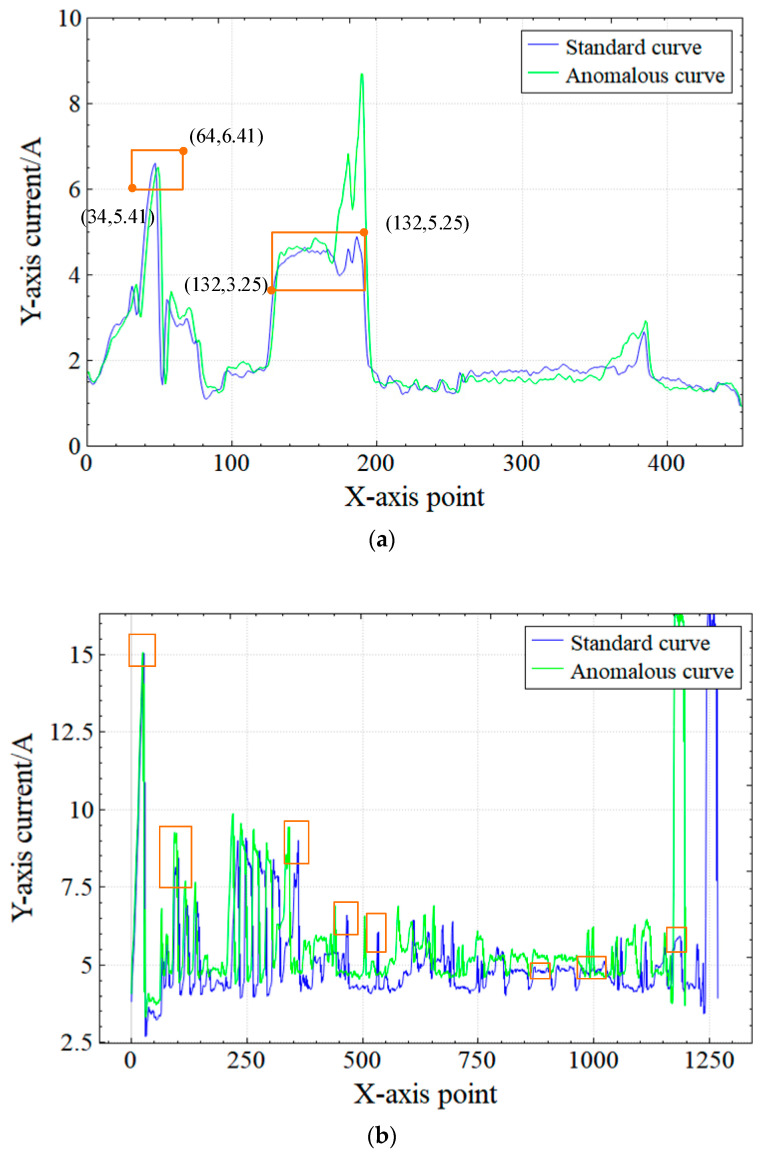
Comparison of standard and anomalous curve: (**a**) Tool-crashing anomaly; (**b**) Override changing anomaly.

Take the process of boring the big end bore of the part of the tool holder in Table 1 as an example, and as shown in Table 5 and Figure 7a, a comparison with the standard curve reveals that there is a steep rise in the curve and numerous data points exceed the bounds of the calibrated characteristic interval, and the average current is greater than 0.1 A. Based on retrospective video verification, the machine was found to have a tool crash. In the case of a car part turning shown in Figure 7b, there is an obvious stretching and rising of the anomalous curve compared with the standard curve. The calibrated feature points and feature intervals only match normally at the beginning of the processing, and all subsequent matches are anomalous. In addition, as shown in Table 6, the anomalous processing time was shortened by 4.4 s, and the current value was increased by 0.5 A. There are 14 consecutive similar cases in the 30 sets of data tested, so it can be confidently judged that the fault is a multiplier anomaly.

## 6. Conclusions

To analyze the characteristics of the total electric current data of the equipment, we propose the use of a feature vector for identifying the operating status of the equipment. In the monitoring process, the electric current data are matched with the feature vector to monitor the production status of the equipment in real-time and to meet the requirements of long-term stable monitoring. The proposed method for monitoring the production status of the processing equipment enables the local storage of a large amount of raw electric current data at the edge, as well as achieving real-time edge computing and status monitoring of production information at the part level, which greatly reduces the computational pressure and storage burden on the central server. It can identify the status of turning, milling, drilling, and boring processes, including power off, processing, tool change, anomaly, and other states. The high-frequency data acquisition and monitoring demands can be satisfied using the proposed monitoring method, which lowers the computing demands of the embedded devices. The proposed monitoring system was tested in the shop during the field processing experiments. The results show that the system can correctly identify the normal and anomalous operational status of multiple heterogeneous equipment. At present, the algorithm can recognize abnormal states, including the tool collision of parts and anomalous magnification of CNC. In the normal operation status, the production throughput can be quantified automatically, and the start time and completion time of each part can be identified. The normal information can be used to count work hours and calculate the performance of employees, while the anomalous information can be used to raise an immediate alarm and halt loss.

This method applies to the real-time monitoring of the operational status of traditional processing equipment and can identify production information and anomalous equipment production statuses. However, the anomalous conditions of actual workshops can be quite diverse. Therefore, the quantitative calculation and identification and qualitative clustering analysis of the anomalous production status of equipment remain a research topic for the future. In this regard, deep learning provides a good solution. In addition, based on the method in this paper, the variety of process features can be further enriched for the part-type identification of mixed-flow machining.

## Figures and Tables

**Table 1:** Part calibration.

No.	Product Name	Process Name	Equipment Name	Loading and Unloading	*I_start_* /A	*I_end_* /A	*T_wait_*	*T_work_*	*I_avg_* /A
1	Toolholder	Turning small end bore	Lathe	Manual	1.20	0.80	100	546	1.64
2	Toolholder	Boring big end bore	Lathe	Manual	1.35	0.70	60	451	2.23
3	DriveShaft	Milling Ballways	Milling Machine	Automatic	1.22	0.95	60	703	1.76
4	DiffCrossShaft	Forging	Forging Machine	Automatic	19.00	18.00	60	80	20.70
5	HUB	Rough turning of end face outer and inner holes	Combination Lathe	Manual	4.66	3.90	100	891	5.84
6	HUB	Fine-turning of end face outer and inner holes	Combination Lathe	Manual	3.54	3.00	100	1095	5.15

**Table 2:** Identification results.

No.	Product Name	Number of Parts	Maximum Processing Time/s	Minimum Processing Time/s	Maximum Wait Time/s	Minimum Wait Time/s	Average Wait Time/s
1	Toolholder	74	35.12	34.51	24.94	7.01	13.80
2	Toolholder	19	28.88	28.69	43.12	13.54	21.76
3	DriveShaft	91	47.02	45.24	15.23	13.98	14.64
4	DiffCrossShaft	408	5.73	4.83	9.88	8.02	9.03
5	HUB	49	57.15	55.40	36.71	11.01	22.17
6	HUB	51	70.11	69.96	20.22	8.62	11.57

**Table 3:** Feature point vectors.

No.	X	Y/A	X-Range	Y-Range/A
1	15	5.70	10	0.5
2	239	5.06	20	0.5
3	663	4.95	20	1.2
4	1446	8.11	20	0.7
5	1887	5.28	20	0.5
6	2614	6.05	20	0.5
7	2809	5.75	20	0.5

**Table 4:** Real-time monitoring results.

No.	Start Time		End Time		Work Time		Wait Time	
	Point Index	Time (h:min:s)	Point Index	Time (h:min:s)	Point Numbers	Duration (s)	Point Numbers	Duration (s)
1	20,134	14:58:10	23,204	15:01:26	3070	196.2	1500	89.9
2	24,704	15:02:55	27,774	15:06:12	3070	196.2	2350	142.1
3	30,124	15:08:35	33,125	15:11:51	3069	196.2	2411	152.2
4	35,536	15:14:13	38,536	15:17:30	3070	196.2	2613	164.2
5	41,149	15:20:04	44,218	15:23:22	3069	197.2	1626	97
6	45,844	15:24:59	48,903	15:28:15	3059	196.2	2359	142.1
7	51,262	15:30:38	54,262	15:33:55	3066	196.2	6503	408.4

**Table 5:** Comparison of tool crash anomaly.

Curve Type	Average Processing Cycle/s	Average Current/A	Number of Workpieces
Standard Curve	28.70	2.24	18
Anomalous Curve	28.79	2.38	1

**Table 6:** Comparison of multiplier anomalies.

Curve Type	Average Processing Cycle/s	Average Current/A	Number of Workpieces
Standard Curve	81.3	5.25	16
Anomalous Curve	76.7	5.75	14

## Data Availability

Not applicable.
